# Modulation of synaptic transmission through O-GlcNAcylation

**DOI:** 10.1186/s13041-023-01072-4

**Published:** 2024-01-02

**Authors:** Seunghyo Han, Jun-Nyeong Kim, Chan Ho Park, Jin-Seok Byun, Do-Yeon Kim, Hyoung-Gon Ko

**Affiliations:** 1 Department of Anatomy and Neurobiology, School of Dentistry, Brain Science and Engineering Institute, Kyungpook National University, 2177 Dalgubeol-daero, Daegu, 41940 South Korea; 2 Department of Dental Biomaterials, School of Dentistry, Kyungpook National University, Daegu, 41940 Republic of Korea; 3 Department of Oral Medicine, School of Dentistry, Kyungpook National University, Daegu, 41940 Republic of Korea; 4 Department of Pharmacology, School of Dentistry, Kyungpook National University, Daegu, 41940 Republic of Korea

**Keywords:** O-GlcNAcylation, O-GlcNAc transferase, O-GlcNAcase, Synaptic transmission, Synaptic plasticity

## Abstract

O-GlcNAcylation is a posttranslational modification where N-acetylglucosamine (O-GlcNAc) is attached and detached from a serine/threonine position by two enzymes: O-GlcNAc transferase and O-GlcNAcase. In addition to roles in diabetes and cancer, recent pharmacological and genetic studies have revealed that O-GlcNAcylation is involved in neuronal function, specifically synaptic transmission. Global alteration of the O-GlcNAc level does not affect basal synaptic transmission while the effect on synaptic plasticity is unclear. Although synaptic proteins that are O-GlcNAcylated are gradually being discovered, the mechanism of how O-GlcNAcylated synaptic protein modulate synaptic transmission has only been reported on CREB, synapsin, and GluA2 subunit of AMPAR. Future research enabling the manipulation of O-GlcNAcylation in individual synaptic proteins should reveal hidden aspects of O-GlcNAcylated synaptic proteins as modulators of synaptic transmission.

## Introduction

Protein properties and activities can be modulated by posttranslational modification (PTM). Addition or removal of specific molecules on amino acid side chains efficiently and reversibly control properties such as binding affinity with other proteins. The most common PTM is phosphorylation while glycosylation, ubiquitination, and SUMOylation also regulate posttranslational protein function. Glycosylation involves covalent attachment of large carbohydrate molecules to a protein. Glycosylation is classified as O-linked, where glycans are covalently bonded to the oxygen of the amino acid side chain, and as N-linked, where glycan is covalently bonded to the nitrogen of the amino acid side chain [1]. O-GlcNAcylation is a recently discovered glycosylation type where N-acetylglucosamine (O-GlcNAc) is added to the hydroxyl oxygen of a serine or threonine side chain by O-GlcNAc transferase (OGT) [2, 3]. Most proteins are processed through glycosylation in the ER to their desired intracellular location, whereas O-GlcNAcylation occurs in the cytoplasm or nucleus. This locational feature of O-GlcNAcylation suggests that other functions are involved besides the simple movement of newly synthesized proteins to specific destinations.

O-GlcNAcylation is similar to phosphorylation in terms of enzyme-mediated chemical reactions. Protein kinases and phosphatases add or remove a phosphate group to a specific amino acid. For O-GlcNAcylation, OGT and O-GlcNAcase (OGA) have similar roles to kinase and phosphatase, respectively [3]. In addition, O-GlcNAc is not randomly attached to an amino acid, and serine or threonine residue are selectively O-GlcNAcylated by OGT. Thus, OGT competes with Ser/Thr kinases on a target protein. Although phosphorylation systems require a variety of target-specific kinases, the regulation of O-GlcNAcylation is only mediated by OGT and OGA. These two enzymes are distributed throughout the body, including the nervous system [4]. Although changes occur in expression with age, OGT and OGA did not show region-specific expression patterns in the brain [4]. Moreover, OGA and OGT are expressed in neurons and in astrocytes and microglia [5]. Dysregulation of O-GlcNAcylation is involved in pathological states such as diabetes, cancer, and neurodegenerative disease. Herein, we discuss O-GlcNAcylation with a focus on synaptic transmission, and consequently, assess the neurophysiological functions of O-GlcNAcylation.

## Two key players for O-GlcNAcylation: OGT and OGA

OGT and OGA are the key enzymes involved in O-GlcNAcylation. For OGT, three isoforms, nucleocytoplasmic (nc), mitochondrial (m) and shortest form (s), are expressed with subcellular localization. The specific role of each isoform is unknown, but their intracellular location may determine their major substrate to a degree. ncOGT and sOGT are located in nucleus and cytosol while mOGT is mainly located in the mitochondria [5]. In the brain, some differences are present dependent on the developmental stage, mOGT is rarely observed and mainly sOGT and ncOGT are expressed [4]. For OGA, alternative splicing produces two spice variants: cytosolic full-length (OGA-FL or L-OGA) and nuclear (OGA-NV or S-OGA). The OGA-FL variant is mainly localized in the nucleus and cytosol, but OGA-NV is exclusively expressed in the mitochondria [6]. As with OGT, OGA shows differences in expression levels depending on the developmental stage. In the brain, the OGA-NV isoform is strongly expressed in the prenatal stage while the OGA-FL isoform is uniformly expressed throughout the life cycle [4]. Neurons have unique structural features that distinguish them from other cells (e.g., axons and dendrites). Interestingly, OGT and OGA are mainly concentrated in pre- and postsynaptic regions, respectively [7]. The biological significance of the synaptic distribution difference of O-GlcNAcylation regulator is currently unknown, and recent studies have focused on the function of O-GlcNAcylated synaptic proteins.

## The effect of O-GlcNAcylation modification in synaptic transmission

OGA/OGT inhibitors or knockout mice have been used to increase or decrease global O-GlcNAcylation level for evaluation in brain slices or cultured neurons. Although consensus has been reached on the effect of O-GlcNAcylation on basal synaptic transmission, the influence on synaptic plasticity remains complex (Table 1) [8–14]. OGA/OGT inhibitor application had no effect on basal synaptic transmission in the CA3-CA1 pathway in hippocampal slices [9, 12]. Furthermore, neither OGA partial deletion (OGA +/−) nor forebrain-specific OGT conditional KO affected basal synaptic transmission in the CA3-CA1 pathway [10, 11]. However, increasing or decreasing O-GlcNAcylation through OGT/OGA inhibitors produced conflicting results on synaptic plasticity. The enhancement of O-GlcNAcylation by OGA inhibitors, such as PUGNAc or thiamet-G, negatively modulates synaptic plasticity, increases long -term potentiation (LTP), and decreases long-term depression (LTD) in the hippocampal CA3-CA1 pathway [8]. However, opposing results were reported where treatment with alloxan, an OGT inhibitor, decreased LTP and where 9d, an OGA inhibitor, treatment increased LTP in the same pathway [9, 12]. Studies in knockout mice appear to support these results showing negative modulation on synaptic plasticity. LTP and LTD were decreased in the hippocampal CA3-CA1 pathway of OGA+/− mice [10], and LTP was enhanced in CA3-CA2 pathway in forebrain-specific OGT conditional KO mice [11]. These conflicting findings regarding the role of O-GlcNAcylation on synaptic plasticity are probably due to the side effects of inhibitors or temporal differences in the manipulation of O-GlcNAcylation levels. Inhibitors acutely suppress OGA or OGT, but deleted effects of *Oga* gene in conventional knockout mice are chronically accumulated. In addition, the use of conventional knockout mice has an issue about developmental effect. Although the manipulation of OGA and OGT tell us a hint for their importance in synaptic transmission, but ultimately, it is more meaningful to study how O-GlcNAcylation of individual proteins affects synaptic transmission. Lastly, a recent study reported that astrocyte-specific OGT in the medial prefrontal cortex (mPFC) O-GlcNacylates glutamate transporter-1 (GLT-1) under stress, which reduces presynaptic release by affecting glutamate uptake through astrocytic GLT-1. However, without stress, astrocyte-specific OGT deletion did not affect synaptic transmission in mPFC [15].

## Function of O-GlcNAcylation of synaptic proteins

**Fig. 1:**
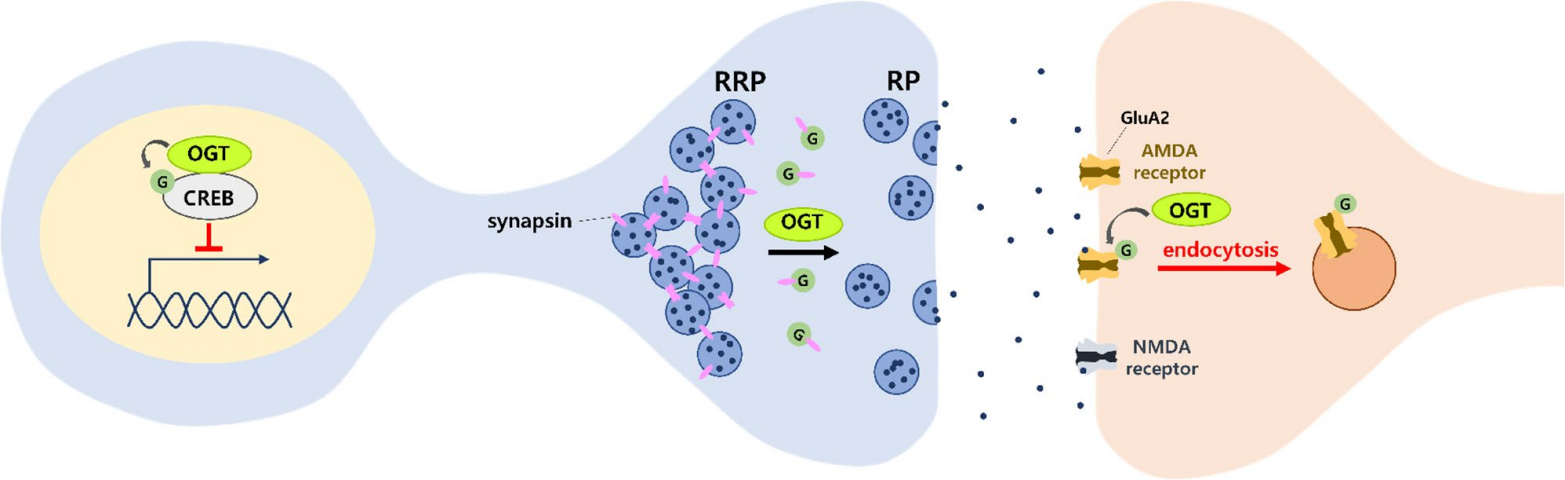
The effects of O-GlcNAcylated proteins on synaptic plasticity. O-GlcNAcylation of CREB suppresses its transcriptional activity. Synapsin is a presynaptic protein located on the synaptic vesicular membrane. Synaptic vesicles are tethered in the RRP zone by a network composed of synapsin and actin. O-GlcNacylated synapsins may facilitate synaptic vesicle movement to the RP zone. Although the exact sites are not identified, O-GlcNAcylation of the GluA2 subunit induces endocytosis of AMPAR. OGT, O-GlcNAc transferase; OGA, O-GlcNAcase; RP, reserve pool; RRP, readily releasable pool

Large-scale proteomics studies have identified many candidate proteins that are O-GlcNAcylated in neurons, specifically in synapses [16–19]. One study have found that 19% of synaptosome proteins are O-GlcNAcylated [17], and identified O-GlcNAcylated synaptic proteins include bassoon, piccolo, shank2, synapsin I, synaptopodin, GKAP, and ankyrin G [16–20]. However, few studies have addressed the specific role of O-GlcNAcylated proteins involved in neuronal function (Fig. 1). This is partially caused by a lack of site-specific O-GlcNAc antibodies and site-directed mutagenesis tool mimicking O-GlcNAcylated or O-GlcNAc-deficient proteins unlike phosphorylation studies. O-GlcNAcylation at S40 suppress the activity of CREB, although this is not a synaptic protein [21, 22]. Site-directed mutation of serine 40 residue to alanine in CREB can mimic O-GlcNAc-deficiency, induced neurite outgrowth, and may activate CREB function as a transcription factor. In addition, the overexpression of S40A CREB in the amygdala facilitated long-term fear memory formation [22]. Synapsin is a presynaptic protein associated with synaptic vesicles that regulates synaptic vesicle release by controlling the movement of synaptic vesicles from the reserve pool (RP) to the readily releasable pool (RRP). In basal conditions, synapsin plays a role in holding synaptic vesicles in the RRP region. Upon action potential, phosphorylated synapsin is detached from SV to induce their movement to RP and subsequent release. Although clear evidence is still lacking, O-GlcNacylation of synapsin at T87 likely induces similar consequence to phosphorylation [23]. The GluA2 is a subunit of AMPAR, an essential postsynaptic voltage-gated channel, for synaptic transmission as well as for synaptic plasticity. Although the exact site on this protein remains unidentified, circumstantial evidence suggests that GluA2 O-GlcNAcylation may induce its endocytosis. Global enhancement of the O-GlcNAcylation level through an OGA inhibitor induced a novel form of LTD based on GluA2 endocytosis [8, 13]. Conversely, OGT inhibitor treatment increases GluA2 surface expression [12]. However, OGT knockout decreases surface GluA2 expression in cultured neurons [24]. Thus, in the absence of a study on the exact position of O-GlcNAcylation on GluA2, investigations on the effect of O-GlcNAcylated GluA2 on synaptic transmission should be approached with caution.

## Conclusions and perspectives

Herein, we discussed how changes in O-GlcNAcylation, i.e., in overall O-GlcNAcylation levels and in O-GlcNAcylation of known individual synaptic proteins, affect synaptic transmission. Although some experimental results using inhibitors or KO mice have implications for the regulation of synaptic transmission by controlling the overall degree of O-GlcNAcylation, these should be interpreted with caution. For example, OGA inhibitor treatment can decrease OGT expression and increase OGA expression because of homeostatic mechanisms [25]. Moreover, OGA inhibition did not unilaterally enhance the O-GlcNAcylation of all individual proteins [26]. Therefore, it is important to study synaptic transmission by manipulating the O-GlcNAcylation of individual synaptic proteins, but as mentioned above, this has technical limitations. Recent advances in synthetic biology have demonstrated the feasibility of technologies that can manipulate only the O-GlcNAcylation of individual target proteins [27]. Therefore, future research on the O-GlcNAcylation of individual synaptic proteins could reveal more details of the regulation of synaptic transmission by O-GlcNAcylated proteins that is currently hidden from view.

**Table 1:** The effects of global alteration of the O-GlcNAcylation level on synaptic transmission

Animal	Brain region	Modulation method	O-GlcNAc level	Basal synaptic transmission	Synaptic plasticity	Note	Reference
rat	CA1, DG (hippocampus)	GlcN&Thiamet-G	+			sIPSC -, mIPSC -, eIPSC -	[14]
	CA1 (hippocampus)	GlcN&Thiamet-G	+			neuronal excitability -	
rat	CA3-CA1 (hippocampus)	glucosamine (GlcN)	+		LTP -, LTD +		[8]
		OGA inhibitor (PUGNAc)	+		LTD +		
		OGA inhibitor (Thiamet-G)	+		LTP -, LTD +		
mouse	CA1 (hippocampus)	OGA inhibitor (Thiamet-G)	+	n.e.	n.e.	neuronal excitability -	[13]
						mEPSC amplitude -	
						GluA2 internalization +	
mouse	CA3-CA1 (hippocampus)	OGA inhibitor (9d)	+	not affected	LTP +	presynaptic release +	[9]
mouse	CA3-CA1 (hippocampus)	conventional OGA +/-	+	not affected	LTP -, LTD -	mEPSC (not affected)	[10]
						AMPA/NMDA ratio (not affected)	
rat	CA3-CA1 (hippocampus)	OGT inhibitor (alloxan)	-	not affected	LTP -	GluA1 and GluA2 surface expression +	[12]
mouse	CA3-CA1 (hippocampus)	OGT inhibitor (alloxan)	-	not affected	LTP -		[9]
mouse	CA3-CA1 (hippocampus)	forebrain specific OGT-cKO	-	not affected	LTP +	NR2A/NR2B +	[11]
					LTD (not affected)	presynaptic release (not affected)	

* +: increase, -: decrease

* n.e.: not examined

## Data Availability

Not applicable. No data was generated during the current study.
